# Serial I-123-FP-CIT SPECT Image Findings of Parkinson's Disease Patients With Levodopa-Induced Dyskinesia

**DOI:** 10.3389/fneur.2018.01133

**Published:** 2018-12-20

**Authors:** Eun Hye Jeong, Mun Kyung Sunwoo, Yoo Sung Song

**Affiliations:** ^1^Department of Neurology, Bundang Jesaeng General Hospital, Daejin Medical Center, Seongnam, South Korea; ^2^Department of Nuclear Medicine, Seoul National University Bundang Hospital, Seongnam, South Korea

**Keywords:** dopamine, I-123-FP-CIT SPECT, levodopa-induced dyskinesia, Parkinson's disease, Parkinson's progression markers initiative

## Abstract

**Background:** Levodopa-induced dyskinesia (LID) is a major complication of dopamine replacement drug usage in Parkinson's disease (PD) patients. Since the mechanism of LID is yet unclear, we analyzed serial [I-123] N-ω-fluoropropyl-2β-carbomethoxy-3β-(4-iodophenyl) nortropane (I-123 FP-CIT) single photon emission computed tomography (SPECT) images. We investigated the changes of dopaminergic innervation during the progression of PD in relation to the development of LID.

**Methods:** Data were obtained from the Parkinson's Progression Markers Initiative (PPMI) database. Two hundred and ninety PD dopamine replacement drug-naïve patients (age 61.0 ± 9.7, M: F = 195: 95) were enrolled. I-123 FP-CIT SPECT images from baseline, 12, 24, and 48 months were analyzed among with clinical factors. specific binding ratios (SBRs) of the striatal regions from I-123 FP-CIT SPECT images were analyzed. We used independent tests and logistic regression for analysis of LID risk association.

**Results:** Among 290 patients, 36 patients developed LID after 48 months follow-up. Baseline MDS-UPDRS Part II and III scores were significantly higher in the PD patients with LID, compared with the PD patients without LID. Striatal SBRs were significantly lower in the PD patients with LID at baseline, 24 and 48 months (*p* < 0.001). Multivariate analysis revealed MDS-UPDRS Part II and putaminal SBRs at baseline and 24 months to be significantly associated with the development of LID (*p* < 0.001). Also, patients who developed LID at 48 months had a higher decrease rate of putaminal SBR at the 24 months (*p* < 0.05), and 48 months (*p* < 0.01) period.

**Conclusion:** In this study, we demonstrated the serial changes of the nigrostriatal dopaminergic innervation in relationship to LID development for the first time. The deterioration rate of dopaminergic innervation was significantly higher in the PD patients who developed LID, compared with the PD patients who did not develop LID. Serial follow up I-123 FP-CIT SPECT acquisition during the course of PD could be helpful in predicting the development of LID.

## Introduction

Parkinson's disease (PD) is a neurodegenerative disorder that is primarily caused by the degeneration of the nigrostriatal pathway (1). It accompanies various motor and non-motor symptoms, due to the depletion of the dopaminergic innervation in the nigrostriatal pathway (2). Currently, dopamine replacement drugs are widely used since they have been effective for symptom alleviation. However, long-term usage of the dopamine replacement drugs eventually leads to a major complication termed as levodopa-induced dyskinesia (LID), presenting as hyperkinetic movements such as dystonia, chorea, and athetosis. levodopa-induced dyskinesia (LID) occurs in up to 50% of patients within 5 years of dopamine replacement drug treatment, and has been a major issue since most patients are eventually affected as the disease progress (3, 4).

While the usage of levodopa in PD patients is inevitable and dopamine replacement drug still remains as the most effective treatment option, there have been numerous studies that have investigated the risk and predictive factors of LID. The dosage of dopamine replacement drugs and age of PD onset were known to be one of the most significant factors for LID development along the progression of PD, increasing the risk over time (5, 6). Additionally, early dopamine drug initiation, cumulative dopamine drug dosage, initial dopamine drug dosage, combination of dopamine agonists, female gender, lower body weight, genetic factors, and smoking history were also previously reported to contribute to the risk for LID development (7–9). There are no validated risk models to predict the possibility of LID development.

Several hypotheses were suggested to explain LID, based on either post-synaptic or pre-synaptic mechanisms (10). The post-synaptic hypothesis focuses on the levodopa induced change of the dopamine receptor sensitivity, which is explained by several mechanisms such as an increase in GABA-biosynthetic enzymes and opioid precursors in the striatal post-synapse (11). On the contrary, the pre-synaptic hypothesis focuses on the relationship between the progression of nigrostriatal denervation and LID (12). Dopamine transporter (DAT) imaging studies localizes pre-synaptic dopamine transporters membrane proteins on the terminals of dopaminergic projections from the substantia nigra to the striatum, thus providing a marker for dopamine terminal innervation. The pre-synaptic hypothesis has been recently supported by several DAT imaging studies that revealed the positive correlation between baseline nigrostriatal dopaminergic denervation and LID development risk (9, 13). Furthermore, serotonergic innervation of the putamen was increased in PD patients with established LID (14), which suggests that serotonergic innervation overtakes the shortage of pre-synaptic striatal dopamine release during LID development (15). However, the mechanisms for LID development is still controversial, with no effective preventatives.

Nigrostriatal denervation worsens as the symptoms of PD progress, but the correlation between nigrostriatal denervation and the development of LID is not clear. While previous studies have revealed the positive association of nigrostriatal degeneration and the risk of LID by analyzing the DAT images, these studies have analyzed the baseline DAT images only (9, 13). We have raised the question whether the decrease of striatal uptake on baseline DAT images contributes to the risk of LID, or merely because the patients who will develop LID were in a more advanced stage of PD at the baseline status. In this study, we have analyzed the baseline and follow-up [I-123] N-ω-fluoropropyl- 2β-carbomethoxy- 3β-(4-iodophenyl) nortropane (I-123 FP-CIT) SPECT images from the Parkinson's Progression Markers Initiative (PPMI) database, to demonstrate whether the risk of LID is truly related with the progression of nigrostriatal degeneration during the course of PD. Additionally, we investigated the possibility of the utilization of follow-up I-123 FP-CIT SPECT images for LID prediction.

## Materials and Methods

### Patients

Data of participants were obtained from the PPMI database (http://www.ppmi-info.org), downloaded in April, 2018. Two ninety PD patients (age 61.0 ± 9.7, M: F = 195: 95) were enrolled. Participants of the PPMI were recruited from 35 centers in North America, Europe, Israel, and Australia. Study participants volunteered to enrollment, and were required to undergo clinical tests and I-123 FP-CIT SPECT imaging. The whole patient group was constituted of 7 Hispanic/Latinos, 2 American Indian/Alaska natives, 3 Asians, 273 Caucasians, and 5 not specified. The inclusion criteria of PPMI were patients with DAT deficit on baseline I-123 FP-CIT SPECT images, a diagnosis of PD for 2 years or less at the time of screening, age 30 years or more, Hoehn and Yahr (H&Y) stage I or II at baseline. Patients were excluded if they were on any kinds of PD related medications. In our study, we narrowed down the PD patients from the PPMI cohort with at least two follow-up I-123 FP-CIT SPECT image (among 12, 24, and 48 months), and an available Movement Disorder Society sponsored Unified Parkinson's Disease Rating Scale (MDS-UPDRS) part IV result at 48 months follow-up. Two hundred and sixty four patients had follow-up I-123 FP-CIT SPECT at 12 and 24 months, and 251 patients had follow-up I-123 FP-CIT SPECT at 48 months. We used the PPMI database of MDS-UPDRS part II and III from the baseline, and part IV from the 48 months follow-up evaluation. The presence of LID was determined based on MDS-UPDRS part IV question 4.1 (score ≥1). Other clinical data such as the average levodopa equivalent daily dose (LEDD) was also obtained. The PPMI study was approved by the local Institutional Review Boards of all participating sites, and written informed consent for clinical and SPECT data were obtained from each participant at enrollment. All subjects gave written informed consent in accordance with the Declaration of Helsinki. All methods were performed in accordance with the relevant guidelines and regulations.

### I-123 FP-CIT SPECT Analysis

For the quality control of image acquisition, processing, and interpretation of multiple institutions, the core imaging lab of PPMI incorporates several qualification processes to maintain standardization and give feedback. In brief, each institution was required to complete an imaging center questionnaire form, undergo a technical set up visit from the core imaging lab, and perform a quality control measurement and scanner calibration with phantom studies. I-123 FP-CIT SPECT scans were performed 4 ± 0.5 h after I-123 FP-CIT injection (111–185 MBq). Images were acquired with a 128 × 128 matrix stepping each 3 degrees for a total of 120 (or 4 degrees for a total of 90) projections with a window of 159 ± 10% KeV. Total scan duration was ~30–45 min. Iterative reconstruction was done without any filtering. Reconstructed files were then transferred to the PMOD software (PMOD Technologies, Zurich, Switzerland), and regions of interest (ROI) were place on the right and left caudate, right and left putamen, and the occipital cortex as a reference tissue. Count densities were extracted and used to calculate specific binding ratios [SBRs, (target region/reference region)-1] for each of the striatal regions. Minimum values between the right and left striatal regions were selected for analysis. Percentage differences between the follow-up SBRs and the baseline SBRs were calculated [%Diff, 100 × (baseline SBR-follow-up SBR)/baseline SBR]. Higher %Diff values between the right and left striatal regions were selected for analysis.

### Statistical Analysis

Analysis was performed with Medcalc version 18.2.1 (MedCalc Software, Belgium). Demographic factors and striatal SBRs between two groups were compared by *t*-test or Mann-Whitney test. Univariate and multivariate analysis for the risk of LID development was done by logistic regression analysis.

## Results

### Demographic Characteristics

Among 290 patients, 36 patients developed LID after 48 months follow-up. The demographic characteristics of the patients with and without LID at the 48 months period are presented in Table 1. Baseline MDS-UPDRS Part II and III scores were significantly higher in the PD patient group who developed LID, compared with the PD patient group who did not develop LID (*p* < 0.001, *p* < 0.01, respectively). No significant differences were observed in age, portion of early-onset PD (EOPD, onset age < 50 years), gender, weight, duration of PD, H&Y stage, usage of dopamine agonists, and LEDD, between the patient group who developed LID and who did not develop LID at the 48 months period.

**Table 1:** Demographic characteristics of PD patients without and with LID development at 48 months follow-up.

	**PD patients without LID at 48 months (*n* = 254)**	**PD patients with LID at 48 months (*n* = 36)**	***p*-value**
Age at PD onset (years)	61.0 ± 9.8	61.0 ± 9.4	0.97
EOPD (*n*, %)	31, 12.2 %	4, 11.1 %	0.85
Gender (Male: Female)	174: 80	21: 15	0.22
Weight (Kg)	82.1 ± 17.3	79.4 ± 17.1	0.39
Duration of Parkinson's disease symptoms until study enroll	23.6 ± 22.2	23.8 ± 18.9	0.94
Hoehn and Yahr staging at baseline	1.5 ± 0.5	1.7 ± 1.5	0.06
MDS-UPDRS Part II score at baseline	6.3 ± 4.0	9.5 ± 4.3	< 0.001
MDS-UPDRS Part III score at baseline	19.9 ± 8.4	23.7 ± 7.8	< 0.01
Use of dopamine agonist, %	63.3	47.2	0.06
Average LEDD at 48 months (g)	1192 ± 1168	1332 ± 863	0.07

*The values presented are the numbers or mean ± standard deviation*.

### I-123 FP-CIT SPECT SBR Analysis

The SBRs of the caudate were significantly lower in the PD group who developed LID compared with the PD group who did not develop LID, at baseline, 12 months follow up, 24 months follow up, and 48 months follow up (*p* < 0.001 for all periods). The SBRs of the putamen were significantly lower in the PD group who developed LID compared with the PD group who did not develop LID, at baseline, 24 months follow up, and 48 months follow up (*p* < 0.001 for all periods). There were no significant differences of putaminal SBR measured at 12 months follow-up between the two groups. SBRs of the putamen and caudate are listed in Table 2.

**Table 2:** SBRs of striatal regions, of PD patients without and with LID development at 48 months follow-up.

	**Caudate**			**Putamen**			
	**Without LID**	**With LID**	***p*-value**	**Without LID**	**With LID**	***p*-value**
Baseline SBR	1.85 ± 0.51	1.46 ± 0.47	< 0.001	0.68 ± 0.22	0.53 ± 0.18	< 0.001
12 months SBR	1.68 ± 0.48	1.32 ± 0.46	< 0.001	0.59 ± 0.19	0.50 ± 0.20	0.07
24 months SBR	1.56 ± 0.50	1.19 ± 0.46	< 0.001	0.56 ± 0.19	0.42 ± 0.16	< 0.001
48 months SBR	1.37 ± 0.48	0.98 ± 0.43	< 0.001	0.48 ± 0.18	0.33 ± 0.17	< 0.001

### Risk Factors for the Development of LID

Univariate analysis identified the factors for LID development at 48 months. MDS-UPDRS Part II (HR 1.18; 95% CI 1.09–1.27), MDS-UPDRS Part III (HR 1.05, 95% CI 1.01–1.10), putaminal SBRs at baseline (HR 0.02; 95% CI 0.00–0.18), putaminal SBRs at 12 months (HR 0.07; 95% CI 0.01–0.55), and putaminal SBRs at 24 months (HR 0.01; 95% CI 0.00–0.12) were significantly associated with the development of LID (Table 3). Putaminal SBRs of 48 months were not analyzed since it would have no value of predicting the risk of LID at 48 months of follow-up.

**Table 3:** Univariate logistic regression analysis for risk factors of LID development at 48 months.

	**HR (95% CI)**	***p*-value**
Age at PD onset (years)	1.00, (0.96–1.04)	0.97
EOPD (age of onset < 50 years)	0.90, (0.30–2.72)	0.85
Female gender	0.80, (0.56–1.15)	0.23
Body weight	0.99, (0.97–1.01)	0.39
Use of dopamine agonist	0.52, (0.26–1.04)	0.07
Hoehn and Yahr staging at baseline	1.93, (0.96–3.87)	0.06
MDS-UPDRS Part II score at baseline	1.18, (1.09–1.27)	< 0.001
MDS-UPDRS Part III score at baseline	1.05, (1.01–1.10)	0.01
Average LEDD at 48 months	1.00, (0.99–1.00)	0.48
Caudate SBR at baseline	0.18, (0.07–0.43)	< 0.001
Caudate SBR at 12 months	0.18, (0.07–0.43)	< 0.001
Caudate SBR at 24 months	0.18, (0.08–0.43)	< 0.001
Putaminal SBR at baseline	0.02, (0.00–0.18)	< 0.001
Putaminal SBR at 12 months	0.07, (0.01–0.55)	< 0.01
Putaminal SBR at 24 months	0.01, (0.00–0.12)	< 0.001

Multivariate analysis with MDS-UPDRS Part II/III, and each putaminal SBRs were also done (Table 4). Among the striatal regions, putaminal SBRs were analyzed due to the more significant hazard ratios compared with caudate SBRs. Within each multivariate analysis, MDS-UPDRS Part II (HR 1.13; 95% CI 1.04–1.23) and putaminal SBR (HR 0.05; 95% CI 0.01–0.42) at baseline, and MDS-UPDRS Part II (HR 1.06; 95% CI 1.01–1.2) at 12 months, and MDS-UPDRS Part II (HR 1.13; 95% CI 1.03–1.24) and putaminal SBR (HR 0.02; 95% CI 0.01–0.27) at 24 months were significantly associated with the development of LID at 48 months.

**Table 4:** Multivariate logistic regression analysis for risk factors of LID development at 48 months.

	**HR (95% CI)**	***p*-value**
MDS-UPDRS Part II score at baseline	1.13, (1.04–1.23)	< 0.01
MDS-UPDRS Part III score at baseline	1.01, (0.97–1.06)	0.63
Putaminal SBR at baseline	0.05, (0.01–0.42)	< 0.01
MDS-UPDRS Part II score at baseline	1.16, (1.01–1.26)	< 0.01
MDS-UPDRS Part III score at baseline	1.00, (0.96–1.06)	0.75
Putaminal SBR at 12 months	0.16, (0.02–1.45)	0.10
MDS-UPDRS Part II score at baseline	1.13, (1.03–1.24)	< 0.01
MDS-UPDRS Part III score at baseline	1.00, (0.96–1.06)	0.74
Putaminal SBR at 24 months	0.02, (0.01–0.27)	< 0.01

### I-123 FP-CIT SPECT for LID Prediction

**Figure 1:**
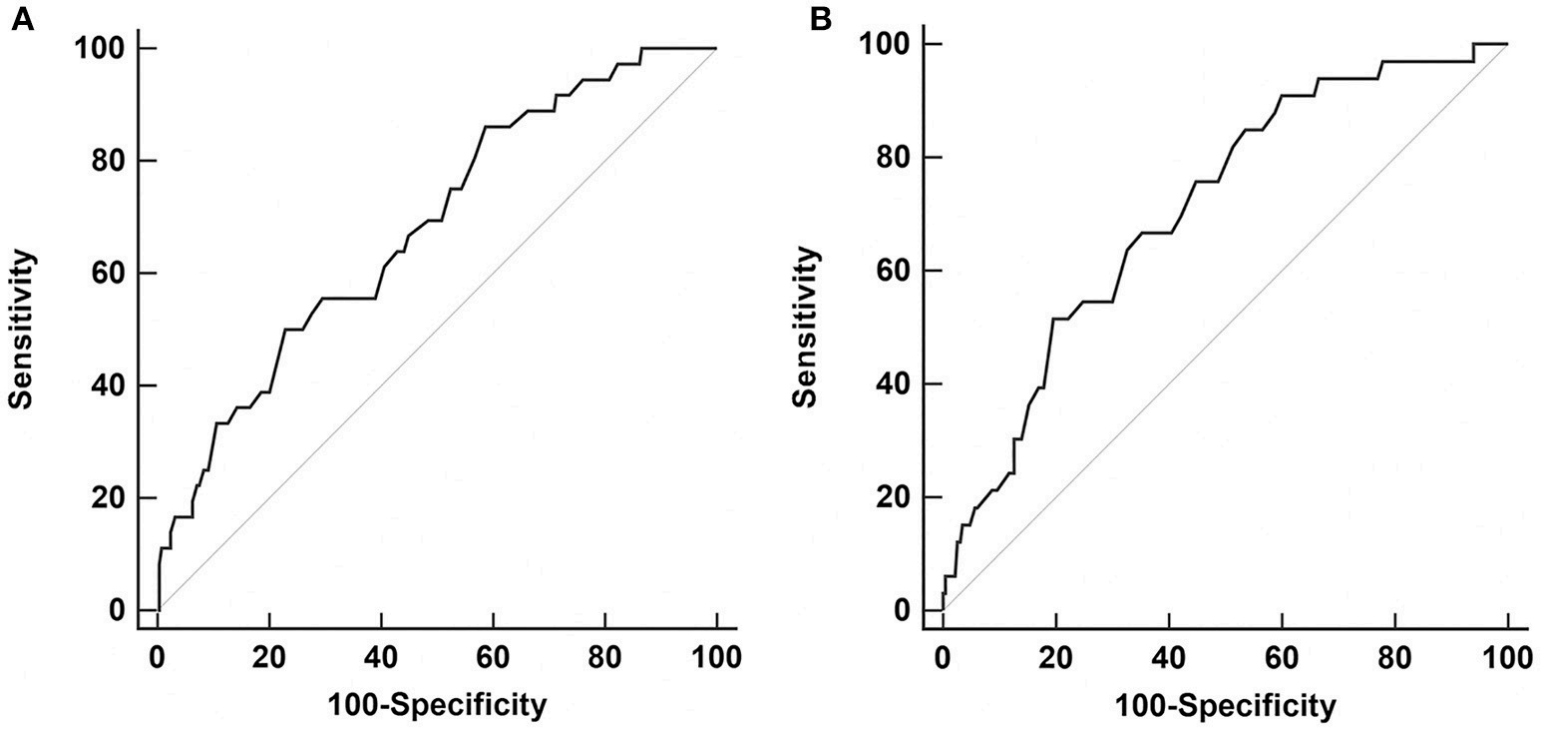
Receiver operating characteristic (ROC) curve analysis for LID development at 48 months, with putaminal SBR values from baseline **(A)** and 24 months **(B)**.

Receiver operating characteristic (ROC) curve analysis with baseline, 12 and 24 months, putaminal SBRs were done for LID development at 48 months. The baseline putaminal SBR with a diagnostic criterion of ≤ 0.68 had a sensitivity of 86.1%, and specificity of 41.3% (*p* < 0.001, area under the curve 0.68). The 24 months putaminal SBR with a diagnostic criterion of ≤ 0.41 had a sensitivity of 51.5% and specificity of 80.4% (*p* < 0.001, area under the curve 0.71) (Figure 1). Comparison of the two ROC curves of the baseline and 24 months showed no significant difference (*p* = 0.25). ROC curve analysis with putaminal SBRs of 12 months were not significant. At 24 months follow-up, only 15 patients among the whole PD group had developed LID.

### Decrease of Putaminal SBRs From Baseline I-123 FP-CIT SPECT

**Figure 2:**
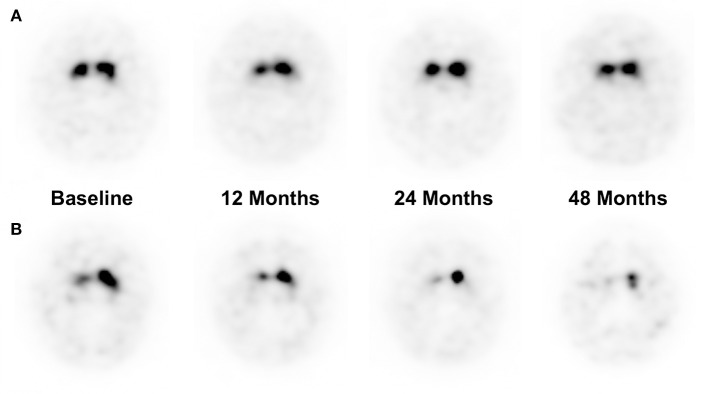
Representative I-123 FP-CIT SPECT images of a patient who did not develop LID at 48 months of follow up **(A)**, and a patient who did develop LID at 48 months **(B)**. **(A)** A 37-years-old male patient had a %Diff of 0, 0, and 10% at 12, 24, and 48 months of follow-up period, respectively. **(B)** A 66-years-old male patient had a %Diff of 23.2, 31.3, 43.8% at 12, 24, and 48 months of follow-up period, respectively.

Percentage Difference of each follow-up time points were obtained, to compare the pace of dopaminergic denervation between the patient groups who developed LID at 48 months of follow-up (Table 5). Patients who developed LID at 48 months had a higher percentage difference at the 24 and 48 months period (*p* < 0.05, *p* < 0.01, respectively), while there were no significant differences of percentage difference at 12 months period. Representative cases of patients who did not develop LID and who did develop LID are illustrated in Figure 2.

**Table 5:** Percentage Difference of putaminal SBRs, from baseline to 12, 24, and 48 months follow-up period.

	**Without LID (%)**	**With LID (%)**	***p*-value**
12 months from baseline	22.2 ± 17.6	23.6 ± 30.4	0.65
24 months from baseline	27.0 ± 20.8	36.4 ± 20.7	< 0.05
48 months from baseline	38.4 ± 20.6	50.4 ± 19.0	< 0.01

## Discussion

To our knowledge, this is the first study to demonstrate the serial changes of nigrostriatal dopaminergic innervation in PD patients during LID development. Along with baseline MDS-UPDRS Part II scores, putaminal SBRs measured with I-123 FP-CIT SPECT at baseline and 24 months were significant risk factors for the development of LID in PD patients, during 48 months of follow-up. The ROC curve analysis revealed that the baseline and 24 months follow-up putaminal SBR were both significant for predicting the development of LID at 48 months. However, in terms of sensitivity and specificity, the two putaminal SBRs implied different clinical values. Putaminal SBRs of the baseline showed a high sensitivity for LID prediction, while the 24 months showed high specificity for LID prediction. The baseline putaminal SBRs may have high sensitivity since patients with low pre-synaptic dopaminergic innervation are more susceptible to levodopa treatment and has a higher risk for LID. However, as the dopaminergic innervation deteriorates in a significant rate in both PD groups with and without LID during the progression of PD, the difference of absolute putaminal SBR values between the two groups would become lower, thus explaining the low sensitivity at 24 months. Patients who have a lower deterioration rate of pre-synaptic dopaminergic innervation at the 24 months would have a lower risk for LID development, thus explaining the higher specificity of putaminal SBRs at 24 months. Therefore, further analysis of the absolute SBR values and deterioration rate during the baseline and 24 months period follow-up could be useful in predicting the risk of LID.

The pre-synaptic hypothesis of LID mainly focuses on the pathologic role of the nigrostriatal pathway. While several other studies have previously demonstrated the relationship between dopaminergic depletion and the development of LID (9, 13), they have only analyzed the baseline F-18 FP-CIT positron emission tomography images, and have not shown the changes of any serial follow-up images during the progression of disease. Therefore, it could not have been concluded whether the decrease of pre-synaptic dopaminergic innervation during the course of disease is truly related with LID risks, or if it is only a prerequisite for LID induction. According to our study, we demonstrated that not only the absolute DAT uptake was decreased in the PD with LID group, but also the progression of dopaminergic denervation was significantly accelerated. Putaminal SBRs at baseline, 24 and 48 months of the PD patient group with LID were significantly lower than those of the PD patient group without LID, despite their similar symptom durations. Difference of SBR values between the two groups were only marginal at 12 months of follow-up. Additionally, %Diff from baseline to 24 and 48 months were also significantly lower. While the striatal neuronal loss during the pre-symptomatic period is known to be decreased in about 30–60% of that of normal controls (16–18), our results suggest that the deterioration rate is also significantly higher in patients who develop LID, during the progression of PD. Since it is known that DAT density and UPDRS motor scores decreases in an exponential pattern during PD progression (19), the decrease rate in the early phase of the PD patients should be higher than in the later phase. In our study, the dopaminergic innervation of the PD group without LID showed a higher decrease rate in the early phase of the disease compared with the later phase, consistent with the exponential decrease model. However, in the PD group with LID, the dopaminergic innervation decreased in a similar rate to the PD group without LID in the early phase but had a higher decrease rate in the later phase of disease. Therefore, according to our findings, we suggest several hypotheses on the development of LID during the progression of PD. The baseline SBR values would reflect the susceptibility to LID development. Normally, the group with lower baseline SBR value would have a lesser decrease of dopaminergic innervation according to the exponential model, but usage of levodopa starts to affect the deterioration rate, resulting in a higher decrease of dopaminergic innervation than expected at 12 months follow-up period. However, the effect of levodopa in the PD group with LID is not sufficient to cause a significant decrease the SBR values at 12 months follow up period, and it seems to take 24 months for levodopa usage to affect the plasticity of dopaminergic innervation, and to accelerate the decrease of dopaminergic innervation. Indices of 12 months follow-up period are not sufficient to reflect these changes. Therefore, we suggest a hypothesis that the baseline SBR values reflect the susceptibility to LID development, while the 24 months follow-up SBR values and %Diff reflect the plasticity to levodopa usage.

The reason for the higher rate of nigrostriatal denervation during the later phase of disease in PD patients with LID compared to the PD patients without LID is not clear, due to the lack of previous studies investigating the pathologic factors that contributes to the loss rate of DAT density and its relationship with LID. There have been several pre-synaptic pathologic factors that were suggested to affect the progression of the striatal neuronal loss from the pre-symptomatic period throughout the progression of PD (17). For example, some PD patients have congenitally low number of baseline striatal neurons due to perinatal neurotoxins or genetic factors. Other compensatory mechanisms such as the plasticity of nigropallidal dopaminergic pathway (20), or serotonergic innervation (21, 22) were also suggested to affect the loss of pre-synaptic striatal neurons during the course of the disease. Such genetic factors or compensatory neuronal plasticity have also been previously reported to affect the development of LID (23, 24). Therefore, future studies investigating the contribution of such pre-synaptic pathologies in dopaminergic denervation and LID development would be needed.

In our study, many other clinical risk factors were included in the analysis, while MDS-UPDRS Part II score and putaminal SBR were the only factors significantly associated with LID development. The Stalevo Reduction in Dyskinesia Evaluation in Parkinson's Disease (STRIDE-PD) study previously identified that young age at onset, higher levodopa dosage, lower body weight, North American geographic region, levodopa agonist usage, female gender, and more severe UPDRS Part II score were high risk factors for LID development (25). Though the UPDRS Part II score was also a consistent significant risk factor in our study, other risk factors had no significance. However, the STRIDE-PD study were conducted with patients who received levodopa treatment from the enroll period, while our patients had a time lag between study enrollment and levodopa treatment. Nonetheless, it was interesting to find that MDS-UPDRS Part II score was associated with LID development, while MDS-UPDRS Part III score was not. This may be because MDS-UPDRS Part II is known to be better in reflecting the deterioration of patient's status over the progression of PD, since levodopa treatment itself may produce motor fluctuations that affects the performance of MDS-UPDRS Part III (26).

We acknowledge several limitations of our study. First, our study does not include the FP-CIT SPECT images of healthy controls, since the PPMI data does not provide follow-up FP-CIT SPECT images nor clinical data of healthy controls. It remains to be seen whether the striatal neuronal loss in PD patients progress in a higher rate compared with those of age and sex matched healthy controls, and whether we can exclude the effect of normal aging process. Second, the PPMI data was collected from multiple institutions, and could have variations in the FP-CIT SPECT image acquisition. In order to maintain a uniformly acquired imaging dataset, quality assurance procedures are performed, as described in the operations manual (www.ppmi-info.org). Third, as mentioned in the methods, all three follow-up FP-CIT SPECT images were acquired in 215 patients out of 290 patients. In 75 patients, only two follow-up FP-CIT SPECT images were acquired. Finally, though we have focused on the pre-synaptic hypothesis for LID development, this does not undermine the post-synaptic hypothesis. Further studies focusing on the post-synaptic striatal signal transduction should be needed.

In conclusion, follow-up FP-CIT SPECT images could be used for the prediction of LID development. Our findings give strength to the pre-synaptic hypothesis for LID development, by showing the rapid pre-synaptic neuronal loss in PD patients who develop LID in a time-dependent manner and necessitates future studies for investigation of the factors affecting the rate of neuronal loss.

## Author Contributions

EJ participated in study conception and design, data collection, statistical analysis, drafting and revising the manuscript. MS and YS contributed to study conception and design, and revising the manuscript.

### Conflict of Interest Statement

The authors declare that the research was conducted in the absence of any commercial or financial relationships that could be construed as a potential conflict of interest.
